# Alternative Plasmonic Materials for Fluorescence Enhancement

**DOI:** 10.1021/acs.jpcc.4c05322

**Published:** 2024-10-22

**Authors:** Stavros Athanasiou, Olivier J. F. Martin

**Affiliations:** Nanophotonics and Metrology Laboratory (NAM), Swiss Federal Institute of Technology Lausanne (EPFL), Lausanne 1015, Switzerland

## Abstract

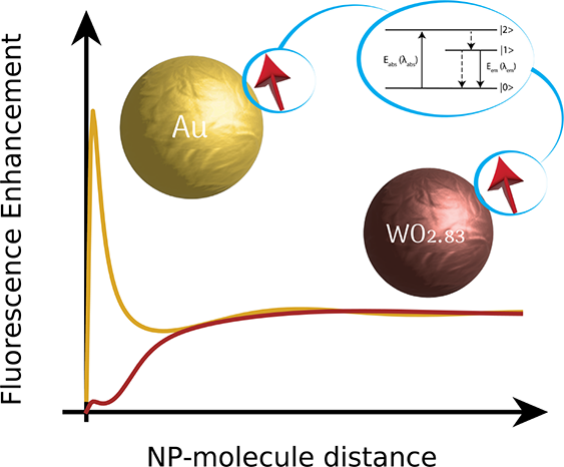

Noble metals such as gold and silver have been used extensively for a range of plasmonic applications, including enhancing the fluorescence rate of a dye molecule, as evidenced by numerous experiments over the past two decades. Recently, a variety of doped semiconductors have been proposed as alternative plasmonic materials, exhibiting plasmonic resonances from ultraviolet to far-infrared. In this work, we investigate the suitability of these alternative materials for enhancing the fluorescence of a molecule. Considering nanosized spheres, we study their response under plane wave illumination and the resulting enhancement factors when coupled to a quantum emitter. Comparisons with standard plasmonic metals reveal that semiconductor materials lead to a significantly reduced, and often strongly quenched, emission of light caused by their dominant absorption, which hinders fluorescence enhancement. However, we show that enhancement may be obtained when considering poor emitting dyes and high refractive index environments. Our findings demonstrate that these alternative materials result in weaker fluorescence enhancement compared to their plasmonic counterparts. Nonetheless, there are means to compensate for this, and a reasonable enhancement can be achieved for dyes in the infrared spectrum.

## Introduction

The modification of the emission rate of a quantum emitter by varying its electromagnetic environment has represented a significant breakthrough to control light–matter interaction.^1^ The enhanced radiative emission rate is fundamentally attributed to the modified local density of states, best described by the Fermi golden rule for quantum transitions.^2,3^ This effect has been realized in several photonic environments^4^ such as in dielectric optical cavities,^5^ photonic crystals,^6,7^ plasmonic nanoparticles,^8,9^ and Mie-resonant particles.^10−13^

In nanometer size particles made from metals with a large free carrier concentration (*n* ∼ 10^23^ cm^–3^), incident light is tightly confined in near-zone regions (so-called hotspots), where light–matter interaction can be enhanced. Noble metals, such as gold, silver, aluminum, and copper, are typically used for plasmonic enhancement due to their potent plasmon resonances and amicable inclination toward nanotechnology.^14−16^

Recently, alternative plasmonic materials—beyond the traditional noble metals—have been proposed, especially classes of semiconducting materials such as metal oxides, nitrides, and chalcogenides, with potential use in electronics, metamaterials, and light-emitting devices.^17−21^ Utilizing these materials for plasmonic applications offers benefits such as flexibility in fabrication and synthesis, as well as the ability to tune the plasmon resonance across the electromagnetic spectrum, from ultraviolet to far-infrared wavelengths.^18^ In contrast, in noble metals, achieving plasmon resonances in the near-infrared spectrum requires either large-size nanoparticles^22,23^ or complex/hybrid geometries.^24,25^

The optical properties of semiconductor nanocrystals, such as the localized surface plasmon resonance and the near-field enhancement, are determined by the dopant type, concentration, and distribution inside the crystal, since these parameters affect indirectly the free carrier concentration, reaching values up to *n* ∼ 10^18^ – 10^21^ cm^–1^.^26^ Consequently, the doping mechanisms allow tuning of plasmon resonances to the infrared spectrum while maintaining small NP sizes and relatively simple geometries.

In this work, we investigate the plasmonic response of semiconducting nanospheres made of the three principal classes: metal oxides, nitrides, and chalcogenides. While their plasmonic responses have been investigated elsewhere,^17,18,27^ we specifically focus on their performances for the fluorescence enhancement of a quantum emitter. This has already been established theoretically and studied experimentally in detail for noble metals, primarily gold.^8,28−36^ Hence, after a brief introduction of the different concepts required for quantifying the modification of the optical properties of a quantum emitter in the presence of a plasmonic nanoparticle in Methods, we study the responses of various plasmonic materials in Results. We review the enhancements obtained with traditional plasmonic metals, such that the responses obtained with alternative materials can be compared to those with plasmonic metals in Discussion. Finally, Conclusions summarizes the key findings of this paper.

## Methods

**Figure 1:**
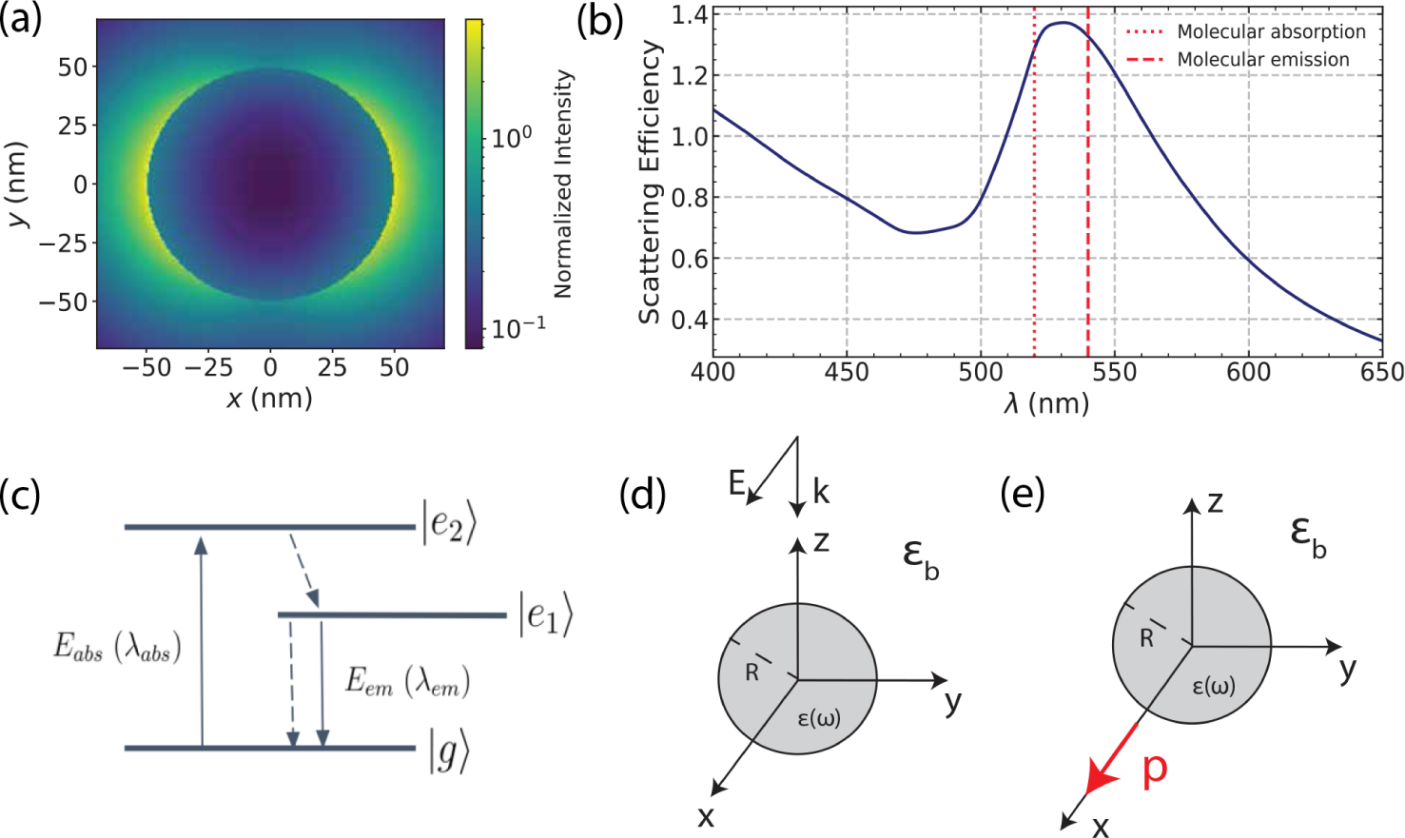
Overview of the physical system. (a) Near-field intensity distribution for a sphere of radius *R* = 50 nm under plane wave illumination. (b) Illustrative spectrum where the plasmon band is in resonance with the molecular absorption λ_abs_ and emission λ_em_. (c) Simple three-level system to model fluorescence in a dye molecule. (d, e) Two-way process for computing the fluorescence enhancement, as described in the main text.

In this section, we briefly describe the modeling approach for light–matter interaction in a system composed of a plasmonic nanoparticle (NP) and a molecule. Our primary focus lies in establishing enhancement factors for comparison with free-space values and thus quantifying the enhancement produced by the NP. Fluorescence is represented by the simple three-level system of Figure 1c, with a nonzero Stokes’ shift. Crucially, both the excitation rate γ_*exc*_ (|g⟩ → |e_2_⟩ transition) and radiative emission rate γ_*rad*_ (|e_1_⟩ → |g⟩ transition) are influenced by parameters that are dependent on the molecule’s environment. Namely, the excitation rate depends on the intensity of the incident electric field, and the emission rate is dictated by the density of optical states (see Methods in the Supporting Information). Thus, we expect both processes to be modified in the presence of a plasmonic NP.

First, for the modification of the excitation rate, the excitation enhancement is defined as the ratio of the excitation rate in the presence of the NP to the value in free space,^29^ i.e.,
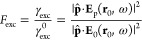
1where *E*_p_(*r*_0_, ω) and *E*_0_(*r*_0_, ω) are the total fields in the presence and absence of the NP at the molecule location ***r***_0_. Note that quantities with the superscript 0 correspond to free-space values from now on.

Then, in the presence of the NP, some of the light emitted by the molecule is scattered by the NP; the corresponding energy can either be emitted as radiation (scattered light) into free space or lost as heat inside the NP (the latter is especially important for plasmonic NPs). The near field enhances the local density of the optical states. To account for these effects, the radiative enhancement factor *F*_rad_ and quenching factor *F*_q_ of the combined system are defined as follows:^37^
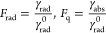
2where γ_rad_ is the total radiative emission rate of the combined system and γ_q_ is the nonradiative rate (or Ohmic losses rate) of the plasmon mode excited in the NP. Then, the modified quantum yield is expressed as the ratio of the new radiative rate to the total rate:
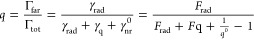
3where *q*^0^ = γ^0^_rad_/(γ^0^_rad_ + γ^0^_nr_) is the intrinsic quantum yield and  the intrinsic nonradiative emission rate. In the absence of the NP, or equivalently when the NP-molecule distance *h* → ∞, we have , γ_q_ → 0, and thus *q* → *q*^0^. For a molecule in free space, the associated fluorescence rate is .^29^ The fluorescence enhancement is defined as
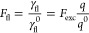
4

Within the dipole approximation, it can be shown that the molecule can be modeled as an oscillating electric dipole placed in the vicinity of a plasmonic NP.^37^ Thus, classical electromagnetic theory can be applied to analyze this system. The radiative enhancement factor can be expressed as the ratio of the emitted power to the far field by the combined system to the power emitted by an isolated dipole, and, likewise, the quenching factor in terms of the absorbed power by the NP is defined as^37^
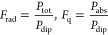
5where *P*_rad_ is the power emitted by the combined system in the far field, *P*_abs_ is the absorbed power in the particle due to Ohmic losses, and *P*_dip_ is the power emitted by a dipole in free space (see Methods in Supporting Information). The modified quantum yield and fluorescence enhancement are given by eqs 3 and 4, respectively.

For our electromagnetic simulations, we use the surface integral equation (SIE) approach.^38,39^ We consider an isolated NP-molecule system in vacuum (*n*_b_ = 1). We chose a simple geometry for the NP, that of a sphere with radius *R* = 50 nm, with a near-field profile shown in Figure 1a. The optimal response of the system is determined by the dielectric function (or the complex refractive index) of the NP material. Refractive index measurements are typically done with ellipsometry using thin films of the material of interest. Unfortunately, the dielectric functions for the alternative plasmonic materials studied here are spread through the literature. To overcome this, we have gathered measured data for all the materials under consideration (see Figures S1–S4, as well as the associated data).^40^

For optimal enhancement, the two characteristic transition energies—absorption and radiative emission (see Figure 1c)—should lie near the plasmon resonance.^35^ Specifically, we choose a spectral configuration in which the wavelengths of the molecular absorption and emission lie symmetrically about the resonance peak with Stokes’ shift (typically found in dye molecules) of λ_stokes_ = λ_em_ – λ_abs_ = 20 nm, as depicted in the example of Figure 1b. Since the objective of our study is to investigate alternative plasmonic materials, we consider a molecule with an intrinsic quantum yield of 1 (*q*^0^ = 1). The effects of nonunity quantum yields are briefly discussed in Discussion.

Since fluorescence entails a 2-step process encompassing both absorption and emission, our computation is split into two parts: (a) a scatterer illuminated with a plane wave, Figure 1d, and (b) the combined system of the scatterer and an electric dipole as the source of radiation, Figure 1e. Step (a) provides the incident field for molecular absorption, which depends on the projection of the electric field onto the molecule dipole moment. Step (b) provides the emitted power to the far-zone by the combined system and the power absorbed by the scatterer, leading to the radiative and nonradiative enhancement factors, as well as the modified yield. From the aforementioned quantities, we can compute the fluorescence enhancement factor. The molecule is placed in one of the hot spots of the nanosphere, according to step (a), with its dipole moment oriented perpendicular to the surface for optimal coupling. For a plane wave propagating along the *z*-direction and polarization along the *x*-direction, the resulting near-field distribution follows that of Figure 1a; thus, optimal enhancement is obtained when the dipole is placed along the *x*-direction. We used this setup throughout our study.

## Results

**Figure 2:**
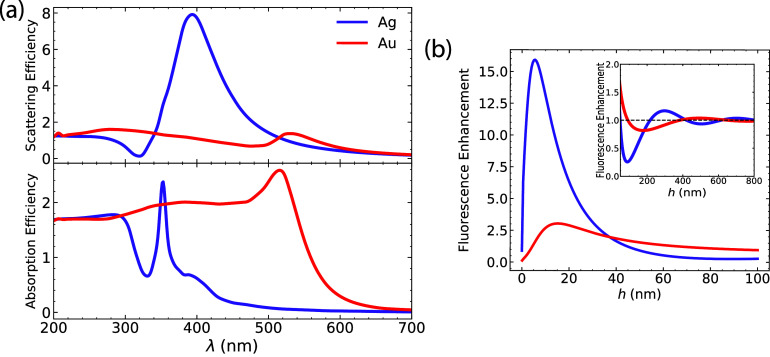
(a) Scattering and absorption efficiencies for gold and silver. (b) The resulting fluorescence enhancement for both metals as a function of the NP-molecule distance *h*. The inset demonstrates an oscillation effect at large distances, whose origin is described in the main text. We use a spherical particle with radius *R* = 50 nm.

As a reference, we address the traditional plasmonic materials gold and silver with localized surface plasmon resonances in the visible spectrum. Among the two, silver exhibits higher light scattering with a plasmon peak at 400 nm as well as lower absorption, as seen in Figure 2a. For a molecule near a silver NP, the fluorescence rate is enhanced by a factor of 16, whereas the enhancement is weaker in the case of the gold NP (fluorescence enhancement by a factor of 3) (Figure 2b). In the silver NP, the excited plasmon mode transfers much of the energy into scattering light localized in the near zone. Both molecular absorption and radiative emission are significantly enhanced, leading to a high fluorescence enhancement. In gold, both molecular optical processes exhibit less enhancement due to decreased scattering and high absorption inside the NP. The latter is reflected from Figure 2b where the NP-molecule distance for optimal enhancement is larger in gold: the molecule has to be a bit further from the surface to overcome quenching.

Examining the inset of Figure 2b, oscillations appear in the fluorescence enhancement as a function of the NP-molecule distance, reminiscent of the interference effect observed when a molecule is positioned in front of a mirror.^41^ The radiation emitted by the dipole is reflected back from the NP and interferes with itself. Depending on the dipole location, we end up in either a minimum or maximum of interference. The amplitude of the reflected wave varies with the absorption of the material; for instance, large absorption results in a weaker reflected wave, and thus, oscillations are feeble. This is precisely the case for gold, while for silver, with its relatively low absorption, oscillations are more prominent.

We turn our attention to semiconductors as alternative plasmonic materials, specifically the three primary classes that have been reported in the literature: metal oxides,^17,27,42−44^ metal nitrides,^17,18^ and metal chalcogenides, in particular copper selenide.^45^

**Figure 3:**
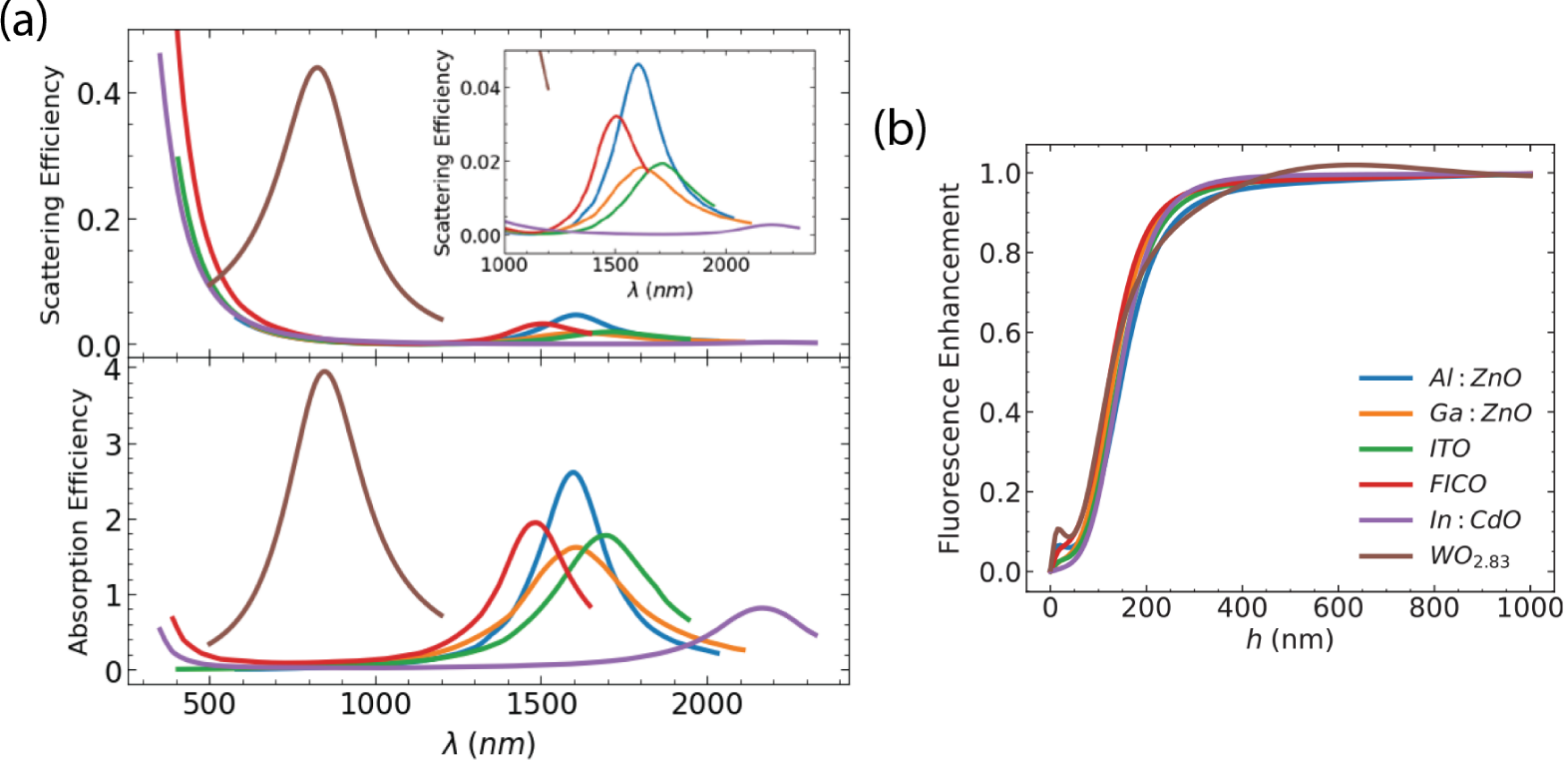
(a) Scattering and absorption spectra for the metal oxides investigated in this study. Absorption is significantly higher than scattering. The inset in the scattering spectra magnifies the plasmon bands for materials exhibiting weak scattering in comparison to that of tungsten oxide. (b) Fluorescence enhancement as a function of the NP-molecule distance *h*. Fluorescence oscillations are entirely suppressed due to high absorption. We use a spherical particle with radius *R* = 50 nm.

In metal oxides, the free carrier concentration is enhanced both intrinsically and extrinsically by conventional techniques. Intrinsic doping is achieved by vacancies in the crystal lattice, typically with oxygen (anion) vacancies, which contribute free electrons. Thus, metal oxides are usually n-type semiconductors. For a nanosphere of *R* = 50 nm, the plasmon resonance lies in the infrared region, as depicted in Figure 3a. Oxides demonstrate relatively weak light scattering and high absorption, as shown in Figure 3a. It is worth noting that among the six oxides, tungsten oxide exhibits the largest light scattering. The combination of weak scattering and strong absorption results in large quenching (Figure 3b). Enhancement is absent in all oxides, except in tungsten oxide, where weak fluorescence enhancement is observed at an NP-molecule distance of *h* ∼ 600 nm, which makes it impractical to implement experimentally.

**Figure 4:**
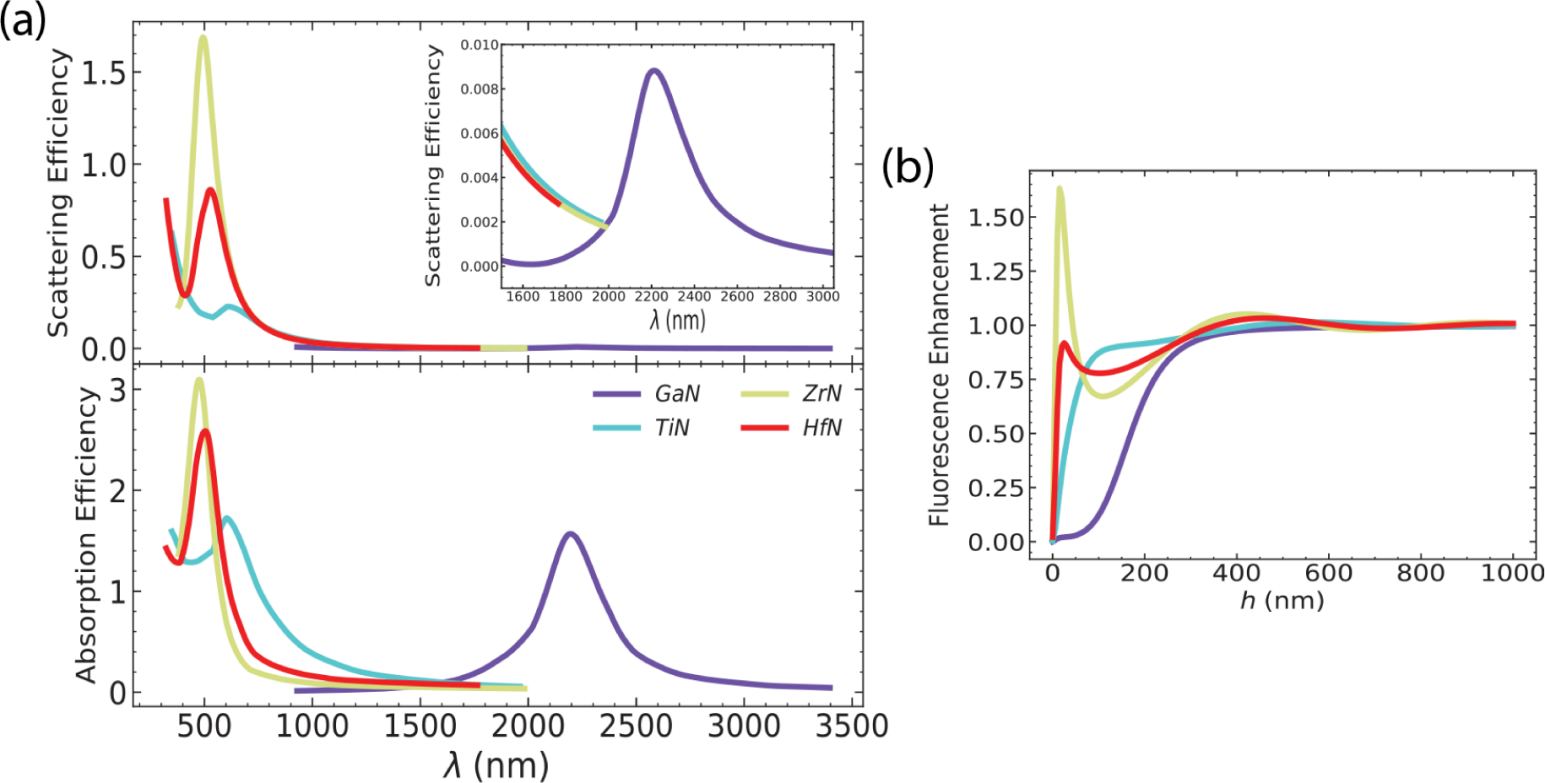
(a) Scattering and absorption efficiencies for a few nitrides. ZrN, TiN, and HfN display a plasmon resonance in the visible spectrum, while GaN displays a plasmon resonance in the far-IR. (b) Fluorescence enhancement as a function of the NP-molecule distance *h*. We use a spherical particle with radius *R* = 50 nm.

Metal nitrides exhibit various states depending on their composition. They can be intrinsically metallic, such as titanium nitride TiN and zirkonium nitride ZrN, or semiconducting, such as gallium nitride GaN.^26^ For those metal-like nitrides, the free carrier concentration is significantly high, comparable to those of gold and silver. The plasmon resonance lies in the visible range (Figure 4a). GaN display a resonance peak in the infrared spectrum and has been considered suitable for tetrahertz optics.^26^ Absorption is significantly larger in GaN compared to light scattering, which leads to an insignificant enhancement, contrary to the rest of the nitrides (Figure 4b). Enhancement is noteworthy in ZrN, with a factor of 1.6 at *h* = 16 nm. A small peak appears in HfN for *h* = 24 nm; however, due to relatively high absorption, it does not exceed the value of 1.

**Figure 5:**
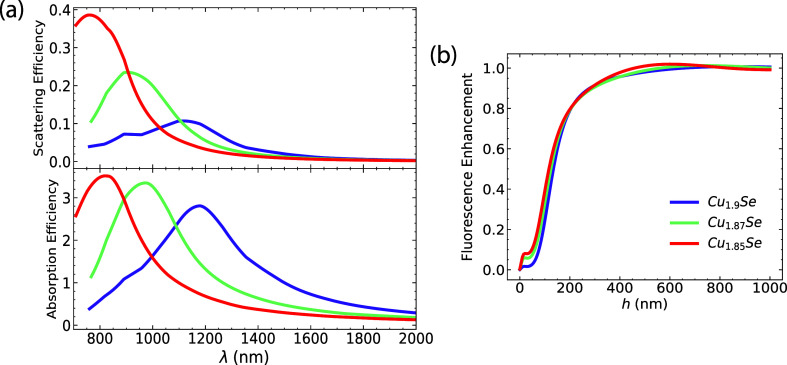
(a) Scattering and absorption efficiencies for copper selenide with different stoichiometries *x*. Increasing doping concentration leads to a blue shift of the plasmon resonance. (b) The resulting fluorescence enhancement as a function of *h*. Quenching dominates over moderate distances, and the overall enhancement is weak. We use a spherical particle with radius *R* = 50 nm.

In doped metal chalcogenides, e.g., Cu_2–*x*_Se and Cu_2–*x*_S, the stoichiometry *x* corresponds to the number of Cu vacancies in the crystal lattice. These cation vacancies are a source of free holes in the crystal and enhance the free carrier concentration, without requiring extrinsic doping.^26^ In Figure 5a, the doping concentration (i.e., the stoichiometry *x*) causes a blue shift in the plasmon resonance. The doping concentration increase further results in stronger resonance peaks, both for scattering and absorption. Higher absorption is attributed to the high rate of free carriers scattering on ionized impurities.^26^ By varying the stoichiometry *x*, the plasmon resonance can be tuned across the infrared spectrum.^45^ In Figure 5b, the fluorescence rate of the molecule is affected negatively at distances of up to 600 nm, where quenching dominates. As is the case with most materials discussed so far, the coupling of the molecule to the plasmonic particle does not lead to significant fluorescence enhancement.

## Discussion

**Figure 6:**
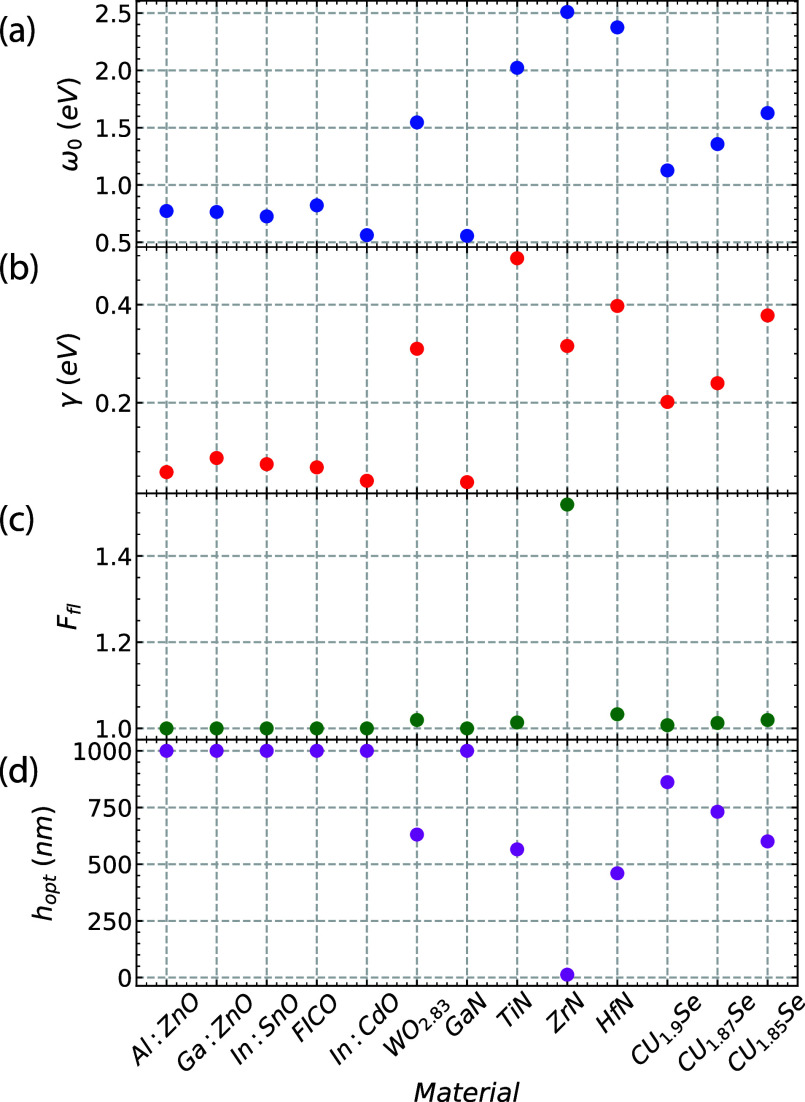
Overview of the main optical characteristics of the considered semiconductor materials. (a) Resonance and (b) fwhm, linked to the intrinsic loss parameter γ, are obtained by fitting the peaks of the scattering spectra with a Lorentzian curve. Resulting optimal values for (c) fluorescence enhancement and (d) the corresponding NP-emitter distance. We use a spherical particle with radius *R* = 50 nm.


Our findings indicate that plasmonic semiconducting materials result in poor fluorescence enhancement compared to their metal counterparts. Figure 6 summarizes the optical properties of the semiconductors and their impact on fluorescence. We can extract useful information about the quality factor of the resonator from the resonance peak of the scattering spectrum. The resonance energy ω_0_ and the resonance width Δω = 2γ are obtained by fitting a Lorentzian function to the plasmon band in the scattering spectrum.

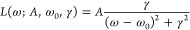
6

where *A* is the amplitude with the proper units. The corresponding values for silver and gold are  and , respectively. We then calculate the quality factor of the resonator, defined as the ratio of the resonance energy to the resonance width *Q* = ω_0_/2_γ_. Thus, for silver, Q^(Ag)^ ≈ 5, and for gold, Q^(Au)^ ≈ 5.7. For the semiconductors, it ranges between *Q*^TiN^ = 2.1 and *Q*^(In:CdO)^ ≈ 7. It appears that the quality factor does not provide a complete picture. While high-quality factors correspond to low damping, explicit computation of the scattering and absorption cross sections reveals that in semiconductors, the latter dominates the overall response and the fluorescence rate is impacted negatively. The complete picture is provided when resorting to the local density of states (LDOS), which provides an alternative definition of the Purcell factor. This approach is particularly suitable for plasmonic systems.^46^ The LDOS consists of contributions from both radiative and nonradiative modes. In semiconductors, the nonradiative contribution outweighs the radiative one, leading to more pronounced quenching in these materials.

Figure 6 also shows that for most materials, the NP-molecule distance for optimal enhancement appears to be beyond applicability since the molecule needs to be placed at distances where quenching is overcome by radiative enhancement. To put everything in perspective, in gold and silver, we have enhancement factors of 3 and 16 at an optimal NP-molecule distance *h* = 15 nm and *h* = 5.5 nm, respectively. Evidently, the reported semiconductors exhibit large absorption, and thus, the resulting fluorescence enhancement is weak, occasionally nonexisting (even for distances up to *h* = 1000 nm). Large absorption stems from the doping process, which on the one side increases the free carrier concentration and thereby accentuates the plasmonic effects. On the other side, doping augments the free carrier concentration by introducing more impurities into the crystal structure, such that electron-impurity scattering becomes significant, contributing to the overall intrinsic damping rate.^26^ It is crucial to highlight that this holds for oxides and copper selenides (or metal chalcogenides in general), albeit to a lesser extent, for nitrides. These results justify the choice of using a relatively large nanosphere since; for smaller sizes, absorption will be even higher, resulting in a further suppression of the enhancement.

Concerning the optical properties of molecules, it is known that within the visible spectrum, dye molecules exhibit notably high, close to unity intrinsic quantum yields.^47^ Yet, as we transition into the infrared spectrum, there is a notable yield decline due to the energy-gap law.^48,49^ This decrease is attributed to an exponential increase in the nonradiative emission rate of the molecule, which occurs as the optical gap energy decreases or, conversely, as the emission wavelength increases. A few illustrative examples of quantum yields for a series of near-IR squaraine dyes include 80% at 681 nm, 75% at 729 nm, 66% at 740 nm, 10% at 820 nm, 0.9% at 885 nm, and 0.8% at 911 nm.^47^ Assuming a fixed molecular absorption cross section, a decreasing yield leads to a greater fluorescence enhancement in the vicinity of an NP, as depicted in Figure S5. Thus, dyes with low intrinsic quantum yields may benefit from some of the semiconducting materials, such as in the case of ZrN and WO_2.83_. It is noteworthy that for very low quantum yields *q*^0^ → 0, fluorescence enhancement is dominated by the excitation and radiative enhancement factors, i.e., *F*_fl_ → *F*_exc_*F*_rad_ since *F*_QY_ (*q*^0^ → 0) → *F*_rad_, while quenching is limited to very small NP-molecule distances *h* → 0, as depicted in Figure S6. The excitation and radiative enhancement factors follow a monotonically decreasing trend, leading to an increased fluorescence enhancement as we move closer to the NP.

While we have performed our simulations using air as the dielectric background, it does not always represent the actual laboratory conditions. Quite often, the NP-molecule systems are synthesized in solvents such as water (*n* = 1.33), ethanol (*n* = 1.36), and toluene (*n* = 1.496), among others. It must be emphasized that there is no universal solvent when it comes to NP synthesis, and the choice depends crucially on the NP material. The different solvents account for a change in the background refractive index, which leads to a red shift of the plasmon peak and an enhanced scattering efficiency, producing an increased fluorescence enhancement (see Figure S7). The enhanced scattering efficiency results from the reduction of the medium’s impedance since *Z* ∼ 1/*n*.

Oscillations may appear in the fluorescence enhancement, as explained in Results. In semiconductors, absorption is relatively large, and the oscillations are weak, resembling the classical system of an overdamped harmonic oscillator. Large absorption in semiconductors also requires larger *h* for optimal fluorescence enhancement.

## Conclusions

We have demonstrated that semiconductor materials, while promising for numerous applications, do not perform as efficiently as traditional plasmonic metals to enhance fluorescence under similar conditions. We find greater plasmonic fluorescence enhancement with the latter ones, while in the former, the magnitude of enhancement varies, generally remaining at lower values. Increasing the free carrier concentration in semiconductors with doping indeed leads to prominent plasmonic effects, such as near-field enhancement. However, this also increases the scattering loss rate, leading to a high absorption, which hinders the enhancement of the fluorescence rate. In fact, at moderate NP-molecule distances, it exerts the opposite effect and quenches the fluorescence. Our work has also highlighted that low intrinsic quantum yields and dielectric environments with a high refractive index can result in a larger enhancement. Consequently, these conditions could boost enhancement in semiconductor NP-molecule systems.

## Data Availability

The data supporting the findings of this work are openly available on Zenodo at 10.5281/zenodo.12623634.

## Supporting Information Available

Dielectric functions of all the materials presented here, the dependence of the fluorescence enhancement on the intrinsic quantum yield and the dielectric environment; additionally, a brief study on the limit of very small intrinsic quantum yields and the impact on the enhancement factors is provided (PDF)

## References

[ref1] Purcell E. M. Spontaneous Emission Probabilities at Radio Frequencies. Phys. Rev. D 1946, 69, 681 10.1103/PhysRev.69.674.2.

[ref2] Loudon R. The Quantum Theory of Light; Oxford University Press, 2000.

[ref3] Shankar R. Principles of quantum mechanics; Springer: New York, NY, 2012.

[ref4] Pelton M. Modified spontaneous emission in nanophotonic structures. Nat. Photonics 2015, 9, 427–435. 10.1038/nphoton.2015.103.

[ref5] Yokoyama H.; Nishi K.; Anan T.; Yamada H.; Brorson S. D.; Ippen E. P. Enhanced spontaneous emission from GaAs quantum wells in monolithic microcavities. Appl. Phys. Lett. 1990, 57, 2814–2816. 10.1063/1.103771.

[ref6] Lodahl P.; Floris Van Driel A.; Nikolaev I. S.; Irman A.; Overgaag K.; Vanmaekelbergh D.; Vos W. L. Controlling the dynamics of spontaneous emission from quantum dots by photonic crystals. Nature 2004, 430, 654–657. 10.1038/nature02772. 15295594

[ref7] Noda S.; Fujita M.; Asano T. Spontaneous-emission control by photonic crystals and nanocavities. Nat. Photonics 2007, 1, 449–458. 10.1038/nphoton.2007.141.

[ref8] Rogobete L.; Kaminski F.; Agio M.; Sandoghdar V. Design of plasmonic nanoantennae for enhancing spontaneous emission. Opt. Lett. 2007, 32, 1623–1625. 10.1364/OL.32.001623. 17572726

[ref9] Russell K. J.; Liu T.-L.; Cui S.; Hu E. L. Large spontaneous emission enhancement in plasmonic nanocavities. Nat. Photonics 2012, 6, 459–462. 10.1038/nphoton.2012.112.

[ref10] Schmidt M. K.; Esteban R.; Sáenz J. J.; Suárez-Lacalle I.; Mackowski S.; Aizpurua J. Dielectric antennas–a suitable platform for controlling magnetic dipolar emission. Opt. Express 2012, 20, 13636–13650. 10.1364/OE.20.013636. 22714428

[ref11] Albella P.; Poyli M. A.; Schmidt M. K.; Maier S. A.; Moreno F.; Sáenz J. J.; Aizpurua J. Low-loss electric and magnetic field-enhanced spectroscopy with subwavelength silicon dimers. J. Phys. Chem. C 2013, 117, 13573–13584. 10.1021/jp4027018.

[ref12] Stamatopoulou P. E.; Tserkezis C. Role of emitter position and orientation on silicon nanoparticle-enhanced fluorescence. OSA Continuum 2021, 4, 918 10.1364/OSAC.412032.

[ref13] Zotev P. G.; Wang Y.; Sortino L.; Severs Millard T.; Mullin N.; Conteduca D.; Shagar M.; Genco A.; Hobbs J. K.; Krauss T. F.; Tartakovskii A. I. Transition metal dichalcogenide dimer nanoantennas for tailored light-matter interactions. ACS Nano 2022, 16, 6493–6505. 10.1021/acsnano.2c00802. 35385647 PMC9047003

[ref14] Jain P. K.; Huang X.; El-Sayed I. H.; El-Sayed M. A. Noble metals on the nanoscale: Optical and photothermal properties and some applications in imaging, sensing, biology, and medicine. Acc. Chem. Res. 2008, 41, 1578–1586. 10.1021/ar7002804. 18447366

[ref15] Zheng J.; Cheng X.; Zhang H.; Bai X.; Ai R.; Shao L.; Wang J. Gold nanorods: The most versatile plasmonic nanoparticles. Chem. Rev. 2021, 121, 13342–13453. 10.1021/acs.chemrev.1c00422. 34569789

[ref16] Abasahl B.; Santschi C.; Raziman T. V.; Martin O. J. F. Fabrication of plasmonic structures with well-controlled nanometric features: A comparison between lift-off and ion beam etching. Nanotechnology 2021, 32, 475202 10.1088/1361-6528/ac1a93. 34348240

[ref17] Naik G. V.; Kim J.; Boltasseva A. Oxides and nitrides as alternative plasmonic materials in the optical range [Invited]. Opt. Mater. Express 2011, 1, 1090 10.1364/OME.1.001090.

[ref18] Naik G. V.; Shalaev V. M.; Boltasseva A. Alternative plasmonic materials: Beyond gold and silver. Adv. Mater. 2013, 25, 3264–3294. 10.1002/adma.201205076. 23674224

[ref19] Kim S.; Kim J.-M.; Park J.-E.; Nam J.-M. Nonnoble-metal-based plasmonic nanomaterials: Recent advances and future perspectives. Adv. Mater. 2018, 30, e1704528 10.1002/adma.201704528. 29572964

[ref20] Hopper E. R.; Boukouvala C.; Asselin J.; Biggins J. S.; Ringe E. Opportunities and challenges for alternative nanoplasmonic metals: Magnesium and beyond. J. Phys. Chem. C 2022, 126, 10630–10643. 10.1021/acs.jpcc.2c01944. PMC9272400 35836479

[ref21] Guo Y.; Xu Z.; Curto A. G.; Zeng Y.-J.; Van Thourhout D. Plasmonic semiconductors: Materials, tunability and applications. Prog. Mater. Sci. 2023, 138, 101158 10.1016/j.pmatsci.2023.101158.

[ref22] Kottmann J. P.; Martin O. J.; Smith D. R.; Schultz S. Spectral response of plasmon resonant nanoparticles with a non-regular shape. Opt. Express 2000, 6, 213–219. 10.1364/OE.6.000213. 19404353

[ref23] Hu M.; Chen J.; Li Z.-Y.; Au L.; Hartland G. V.; Li X.; Marquez M.; Xia Y. Gold nanostructures: Engineering their plasmonic properties for biomedical applications. Chem. Soc. Rev. 2006, 35, 1084–1094. 10.1039/b517615h. 17057837

[ref24] Zhang W.; Gallinet B.; Martin O. J. F. Symmetry and selection rules for localized surface plasmon resonances in nanostructures. Phys. Rev. B 2010, 81, 233407 10.1103/PhysRevB.81.233407.

[ref25] Ayala-Orozco C.; Liu J. G.; Knight M. W.; Wang Y.; Day J. K.; Nordlander P.; Halas N. J. Fluorescence enhancement of molecules inside a gold nanomatryoshka. Nano Lett. 2014, 14, 2926–2933. 10.1021/nl501027j. 24738706 PMC4023845

[ref26] Agrawal A.; Cho S. H.; Zandi O.; Ghosh S.; Johns R. W.; Milliron D. J. Localized Surface Plasmon Resonance in Semiconductor Nanocrystals. Chem. Rev 2018, 118, 3121–3207. 10.1021/acs.chemrev.7b00613. 29400955

[ref27] West P. R.; Ishii S.; Naik G. V.; Emani N. K.; Shalaev V. M.; Boltasseva A. Searching for better plasmonic materials. Laser Photon. Rev. 2010, 4, 795–808. 10.1002/lpor.200900055.

[ref28] Kühn S.; Håkanson U.; Rogobete L.; Sandoghdar V. Enhancement of Single-Molecule Fluorescence Using a Gold Nanoparticle as an Optical Nanoantenna. Phys. Rev. Lett. 2006, 97, 017402 10.1103/PhysRevLett.97.017402. 16907406

[ref29] Anger P.; Bharadwaj P.; Novotny L. Enhancement and Quenching of Single-Molecule Fluorescence. Phys. Rev. Lett. 2006, 96, 113002 10.1103/PhysRevLett.96.113002. 16605818

[ref30] Muskens O. L.; Giannini V.; Sanchez-Gil J. A.; Gómez Rivas J. Strong enhancement of the radiative decay rate of emitters by single plasmonic nanoantennas. Nano Lett. 2007, 7, 2871–2875. 10.1021/nl0715847. 17683156

[ref31] Ringler M.; Schwemer A.; Wunderlich M.; Nichtl A.; Kürzinger K.; Klar T. A.; Feldmann J. Shaping Emission Spectra of Fluorescent Molecules with Single Plasmonic Nanoresonators. Phys. Rev. Lett. 2008, 100, 203002 10.1103/PhysRevLett.100.203002. 18518528

[ref32] Baffou G.; Girard C.; Dujardin E.; Colas des Francs G.; Martin O. J. F. Molecular quenching and relaxation in a plasmonic tunable system. Phys. Rev. B 2008, 77, 121101 10.1103/PhysRevB.77.121101.

[ref33] Bardhan R.; Grady N. K.; Cole J. R.; Joshi A.; Halas N. J. Fluorescence enhancement by Au nanostructures: Nanoshells and nanorods. ACS Nano 2009, 3, 744–752. 10.1021/nn900001q. 19231823

[ref34] Kinkhabwala A.; Yu Z.; Fan S.; Avlasevich Y.; Müllen K.; Moerner W. E. Large single-molecule fluorescence enhancements produced by a bowtie nanoantenna. Nat. Photonics 2009, 3, 654–657. 10.1038/nphoton.2009.187.

[ref35] Kern A. M.; Meixner A. J.; Martin O. J. F. Molecule-dependent plasmonic enhancement of fluorescence and Raman scattering near realistic nanostructures. ACS Nano 2012, 6, 9828–9836. 10.1021/nn3033612. 23020510

[ref36] Zhao W.; Tian X.; Fang Z.; Xiao S.; Qiu M.; He Q.; Feng W.; Li F.; Zhang Y.; Zhou L.; et al. Engineering single-molecule fluorescence with asymmetric nano-antennas. Light: Sci. Appl. 2021, 10, 79 10.1038/s41377-021-00522-9. 33854033 PMC8046762

[ref37] Novotny L.; Hecht B. Principles of nano-optics, 2nd ed.; Cambridge University Press: Cambridge, England, 2012.

[ref38] Kern A. M.; Martin O. J. F. Surface integral formulation for 3D simulations of plasmonic and high permittivity nanostructures. J. Opt. Soc. Am. A 2009, 26, 732–740. 10.1364/JOSAA.26.000732. 19340246

[ref39] Raziman T. V.; Somerville W. R. C.; Martin O. J. F.; Ru E. C. L. Accuracy of surface integral equation matrix elements in plasmonic calculations. J. Opt. Soc. Am. B 2015, 32, 485–492. 10.1364/JOSAB.32.000485.

[ref40] Athanasiou S.; Martin O. J. F. Alternative plasmonic materials for fluorescence enhancement [Data set]; Zenodo, 2024. 10.1021/acs.jpcc.4c05322 PMC11533197 39502803

[ref41] Chance R. R.; Prock A.; Silbey R. Lifetime of an excited molecule near a metal mirror: Energy transfer in the Eu3+/silver system. J. Chem. Phys. 1974, 60, 2184–2185. 10.1063/1.1681335.

[ref42] Manthiram K.; Alivisatos A. P. Tunable localized surface plasmon resonances in tungsten oxide nanocrystals. J. Am. Chem. Soc. 2012, 134, 3995–3998. 10.1021/ja211363w. 22332881

[ref43] Gordon T. R.; Paik T.; Klein D. R.; Naik G. V.; Caglayan H.; Boltasseva A.; Murray C. B. Shape-dependent plasmonic response and directed self-assembly in a new semiconductor building block, indium-doped cadmium oxide (ICO). Nano Lett. 2013, 13, 2857–2863. 10.1021/nl4012003. 23701224

[ref44] Ye X.; Fei J.; Diroll B. T.; Paik T.; Murray C. B. Expanding the spectral tunability of plasmonic resonances in doped metal-oxide nanocrystals through cooperative cation-anion codoping. J. Am. Chem. Soc. 2014, 136, 11680–11686. 10.1021/ja5039903. 25066599

[ref45] Dorfs D.; Härtling T.; Miszta K.; Bigall N. C.; Kim M. R.; Genovese A.; Falqui A.; Povia M.; Manna L. Reversible tunability of the near-infrared valence band plasmon resonance in Cu(2-x)Se nanocrystals. J. Am. Chem. Soc. 2011, 133, 11175–11180. 10.1021/ja2016284. 21728384

[ref46] Benisty H.; Greffet J.; Lalanne P. Introduction to Nanophotonics, In Oxford graduate texts; Oxford University Press, 2022.

[ref47] Mayerhöffer U.; Gsänger M.; Stolte M.; Fimmel B.; Würthner F. Synthesis and molecular properties of acceptor-substituted squaraine dyes. Chemistry 2013, 19, 218–232. 10.1002/chem.201202783. 23180571

[ref48] Englman R.; Jortner J. The energy gap law for radiationless transitions in large molecules. Mol. Phys. 1970, 18, 145–164. 10.1080/00268977000100171.

[ref49] Jang S. J. A simple generalization of the energy gap law for nonradiative processes. J. Chem. Phys. 2021, 155 (16), 164106 10.1063/5.0068868. 34717346

