# Supplementary material for: Alternative Plasmonic Materials for Fluorescence Enhancement
Source: J Phys Chem C Nanomater Interfaces. 2024 Oct 22;128(43):18574–81. doi: 10.1021/acs.jpcc.4c05322 (PMC11533197; doi:10.1021/acs.jpcc.4c05322)
Supplement: Supplementary file 1 — jp4c05322_si_001.pdf [file jp4c05322_si_001.pdf]

Alternative Plasmonic Materials for Fluorescence Enhancement

Stavros Athanasiou* and Olivier J.F. Martin*

Nanophotonics and Metrology Laboratory (NAM), Swiss Federal Institute of Technology Lausanne (EPFL), 1015 Lausanne, Switzerland

E-mail: stavros.athanasiou@epfl.ch; olivier.martin@epfl.ch

Supporting Information on Methods

We offer additional details on the physical system and the methodology described in the Methods section of the manuscript.

We consider an isolated molecule in free space at position $\mathbf{r}_0$, illuminated by light with frequency ω. We adopt a semiclassical approach, in which we treat light as a classical electromagnetic wave and matter as a quantum system with its dynamics governed by the Schrödinger equation.[1,2] For weak illumination, the time-dependent perturbation theory is employed to derive the probability rate of quantum mechanical transitions from an initial state $|i\rangle$ to a final state $|f\rangle$, known as the Fermi golden rule.

A typical molecule exhibits a variety of processes, including optical absorption, radiative emission in the form of fluorescence and phosphorescence and nonradiative emission. We restrict ourselves to fluorescence, represented by a simple three-level system with a nonzero Stokes shift. If the size of the molecule is much smaller than the wavelength of the incoming light, the electronic transition can be treated in the dipole approximation, and thus the perturbation takes the form of $H' = -\mathbf{p} \cdot \mathbf{E}(\mathbf{r}_0, \omega)$ with $\mathbf{p} = |p|\hat{\mathbf{p}}$, resembling the potential

energy of a classical electric dipole in an electric field.

For the absorption (or excitation) process, we employ Fermi's golden rule,[2]

$$\gamma_{exc} = \frac{2\pi}{\hbar}|\langle g|H'|e\rangle|^2\delta(E_e - E_g - \hbar\omega) = \frac{\alpha}{\hbar\omega}|\hat{\mathbf{p}}\cdot\mathbf{E}(\mathbf{r}_0,\omega)|^2\,, \tag{1}$$

where α is the molecular absorption cross section and depends on the transition dipole moment $\mathbf{p} = q\langle g|\mathbf{r}|e\rangle$. The absorption cross section is a property of the molecule and can be determined experimentally.[3] The transition rate crucially depends on the projection of the incident field $\mathbf{E}(\mathbf{r}_0,\omega)$ along the direction of the dipole moment $\hat{\mathbf{p}}$. For a randomly-oriented molecule, one may average over all the possible dipole moment orientations.

The non-radiative relaxation process to a lower-energy excited state $|e_2\rangle$ happens faster than a radiative transition back to the ground state $|g\rangle$, i.e. the optical spectrum of the molecule exhibits a Stokes shift. The process of spontaneous radiative emission, corresponding to the transition $|e_2\rangle \to |g\rangle$, occurs at a larger wavelength or, equivalently, smaller energy compared to absorption[4]

$$\gamma^0_{rad} = \frac{\pi\omega}{3\hbar\varepsilon_0}|\mathbf{p}|^2\rho(\mathbf{r}_0,\hbar\omega)\,, \tag{2}$$

where $\rho(\mathbf{r}_0,\hbar\omega)$ is the local density of optical states, evaluated at the position of the molecule. We determine the radiation efficiency of a molecule by defining the intrinsic quantum yield $q^0 = \gamma^0_{rad}/(\gamma^0_{rad} + \gamma^0_{nr})$ where γ^0_{nr} is the non-radiative emission rate. We note that quantities with the superscript 0 correspond to free-space values.

In the presence of the nanoparticle, both absorption and emission of the molecule are modified. We quantify this effect by defining enhancement factors as emphasized in the manuscript

$$F_{exc} = \frac{\gamma_{exc}}{\gamma^0_{exc}}, \quad F_{rad} = \frac{\gamma_{rad}}{\gamma^0_{rad}}, \quad F_q = \frac{\gamma_{abs}}{\gamma^0_{rad}}\,, \tag{3}$$

and the fluorescence enhancement and modified yield are given by

$$F_{fl} = F_{exc}\frac{q}{q^0}, \qquad \frac{q}{q^0} = \frac{F_{rad}}{F_{rad} + F_q + \frac{1}{q^0} - 1}\frac{1}{q^0}\,. \tag{4}$$

The fluorescence enhancement approaches unity as $h \to \infty$. Since $\gamma_{em} = 1/\tau$, then the inverse of F_{fl} gives the fluorescence lifetime enhancement i.e. $F_\tau = \tau/\tau^0 = 1/F_{fl}$.

We adopt a mere classical electromagnetic approach for the radiative emission process, since light-matter interaction is weak (thus the molecular energy landscape is not modified by the NP presence[5]). Within the dipole approximation, the quantum system is modelled as an oscillating electric dipole placed in the vicinity of a plasmonic NP. It must be emphasized that we avoid regimes where the laser power becomes important, the so-called saturation regime,[6] and regimes where non-local quantum effects, electron spilling and Landau damping, become important such as in very small NPs and for very small NP-emitter distance.[7,8] The radiative enhancement factor can be expressed as the ratio of the emitted power to the far-field by the combined system to the power emitted by an isolated dipole, and, likewise, the quenching factor in terms of the absorbed power by the NP,[4]

$$F_{rad} = \frac{P_{tot}}{P_{dip}}\,, \quad F_q = \frac{P_{abs}}{P_{dip}}\,, \tag{5}$$

where P_{tot} is the power emitted by the combined system, P_q the absorbed power in the particle and P_{dip} is the power emitted by a single dipole in free space. The power emitted by the combined system is given by the surface integral of the time-averaged Poynting vector in the far zone; for practical reasons, we perform this over a large sphere of radius $R' = 10\mu m$. Thus, in the far zone we obtain

$$P_{tot}(\omega) = \frac{1}{2Z}\int_A dA\, |\mathbf{E}_{tot}(\mathbf{r},\omega)|^2\,, \tag{6}$$

where Z is the impedance of the surrounding medium, which we will assume to be air,

$Z = \sqrt{\mu_0/\epsilon_0} \simeq 377\,\Omega$, and $\mathbf{E}_{tot}(\mathbf{r},\omega)$ is the total field, i.e. the sum of the field scattered off the NP and the field of the dipole source. The absorbed power accounts for the Ohmic losses in the NP,[9]

$$P_{abs}(\omega) = \frac{1}{2}\int_V dV\ Re\{\sigma\}|\mathbf{E}_V(\mathbf{r},\omega)|^2\,, \tag{7}$$

where $\sigma = -i\omega\varepsilon_0\chi = -i\omega\varepsilon_0(\varepsilon_r - 1)$ is the conductivity of the material, ε_r is the relative dielectric function of the material, V is the volume of the scatterer and $\mathbf{E}_V(\mathbf{r},\omega)$ is the field inside the scatterer.

From the same definitions, we can also compute the scattering and absorption spectra obtained by plane wave illumination on the plasmonic particle (without the dipole). The scattering (absorption) cross section is defined as $C_{sca(abs)} = P_{sca(abs)}/I_{inc}$ where $I_{inc} = \frac{1}{2}\varepsilon_0 c|\mathbf{E}_{inc}|^2$ is the intensity of the incident plane wave, ε_0 is the vacuum permittivity and c the speed of light in vacuum. The scattering (absorption) efficiency is taken as the ratio of the corresponding cross section to the physical one, i.e. $Q_{sca(abs)} = C_{sca(abs)}/\pi R^2$ for the case of a spherical scatterer with radius R.

Supplementary Results

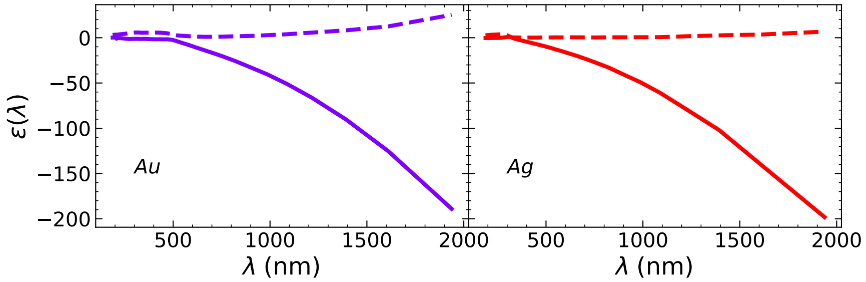

Figure S1. Dielectric function for the metals investigated in this work[10] . The solid (dashed) line corresponds to the real (imaginary) part of the dielectric function.

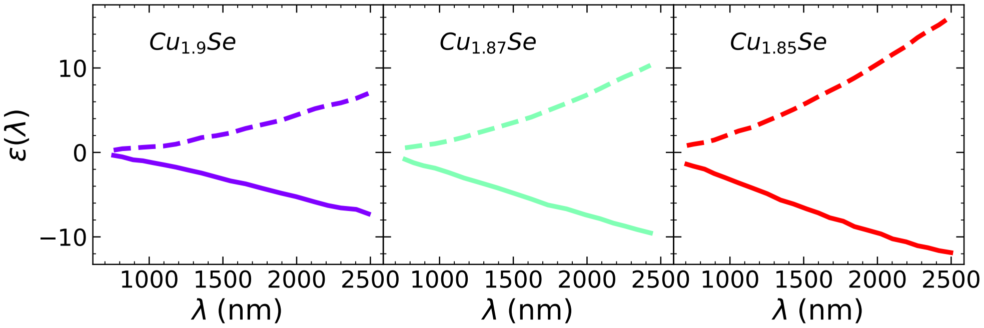

Figure S2. Dielectric function for the metal chalcogenides investigated in this work[11] . The solid (dashed) line corresponds to the real (imaginary) part of the dielectric function.

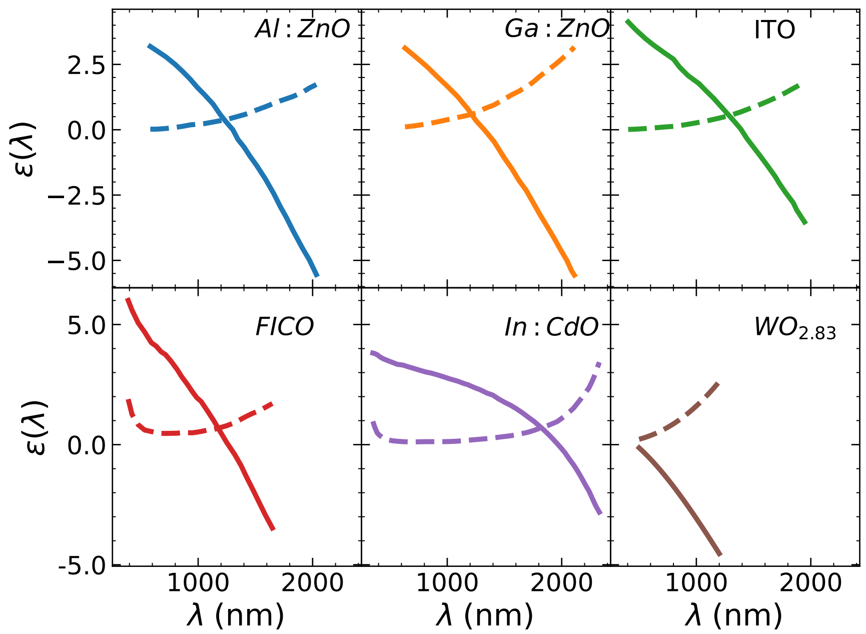

Figure S3. Dielectric function for the oxides investigated in this work[12–16]. The solid (dashed) line corresponds to the real (imaginary) part of the dielectric function.

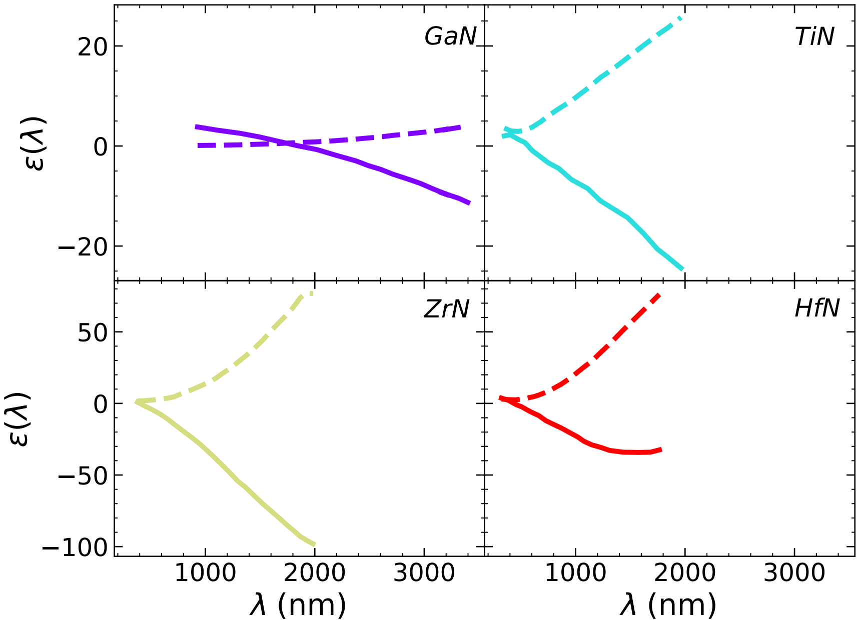

Figure S4. Dielectric function for the nitrides investigated in this work[13,17]. The solid (dashed) line corresponds to the real (imaginary) part of the dielectric function.

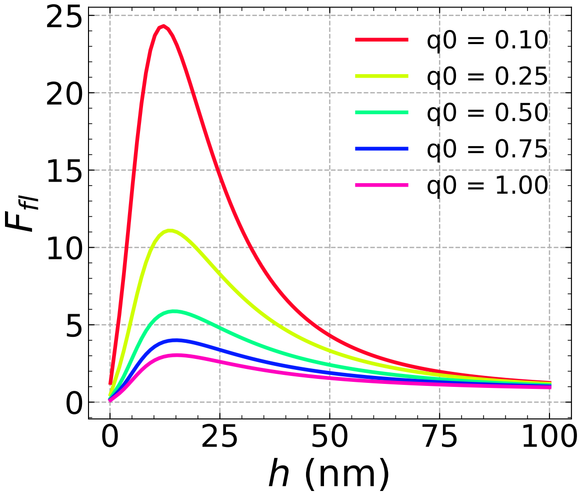

(a) Au

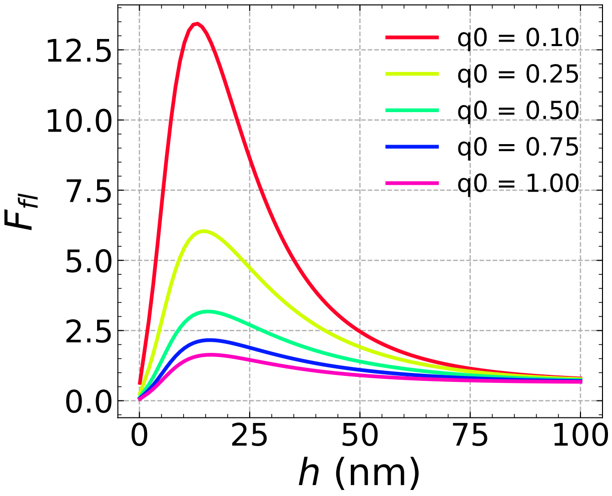

(b) ZrN

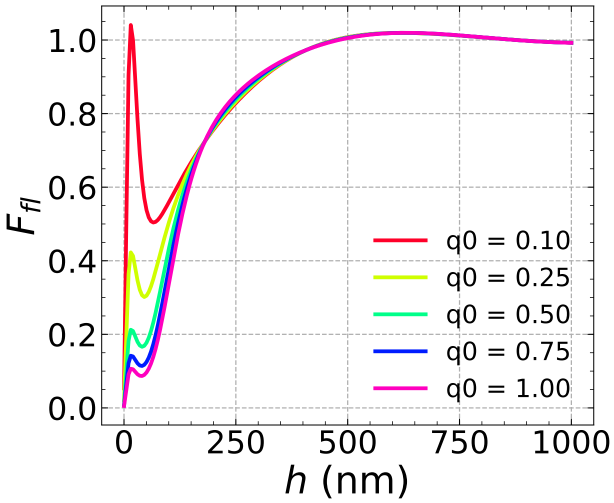

(c) $WO_{2.83}$

Figure S5. Fluorescence enhancement as a function of the NP-molecule distance h for selected materials and intrinsic quantum yields.

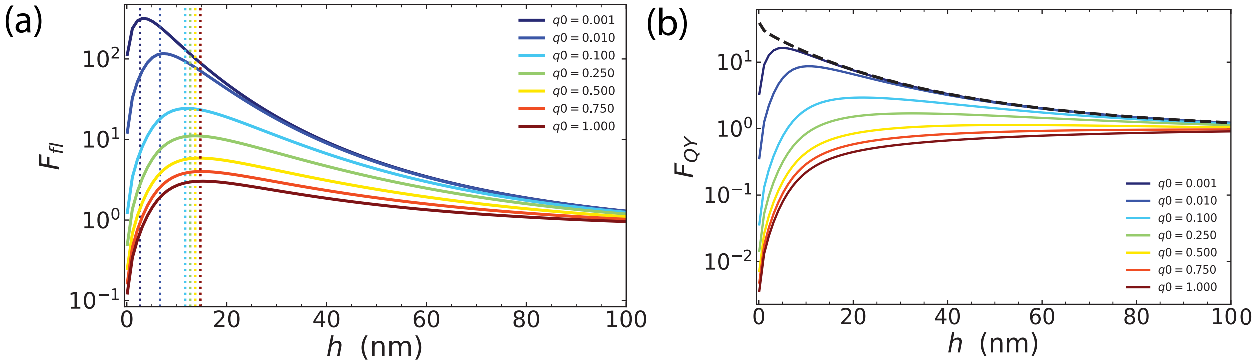

Figure S6. (a) Fluorescence enhancement factor as a function the NP-molecule distance h and the intrinsic quantum yield q_0. The dotted lines indicate the position of optimal enhancement. As the yield reduces, the optimal position shifts closer to the nanoparticle. (b) Quantum yield enhancement $F_{QY} = q/q^0$ as a function of the NP-molecule distance h for various values of the yield. The dashed black line corresponds to the radiative enhancement factor F_{rad}. It is shown that for very small yields, the emission enhancement approaches F_{rad}, as discussed in the main text.

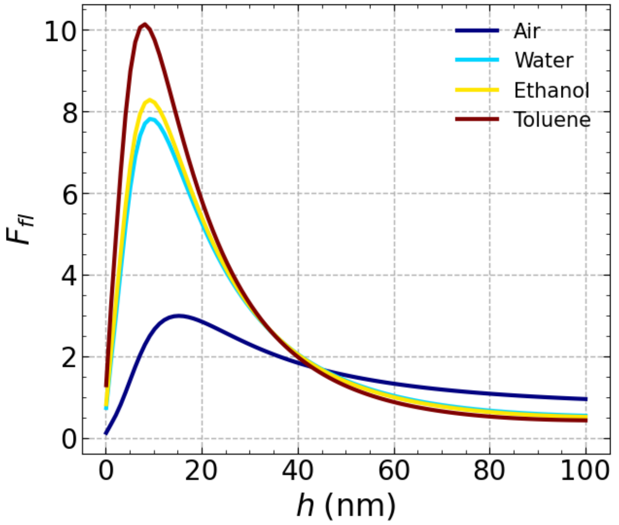

Figure S7. Dependence of the fluorescence enhancement factor on the dielectric environment. The enhancement increases with the refractive index n (or equivalently the dielectric constant $\varepsilon_r = n^2$) of the surrounding medium. This is attributed to the fact that the scattering of light to the far-field is stronger since the impedance of the medium decreases with the refractive index, i.e. $Z = \sqrt{\mu/\varepsilon} = \sqrt{\mu_0/\varepsilon_r\varepsilon_0} \sim 1/n$ where we used $\mu_r = 1$. The refractive indices for the solvents used here are $n = 1$ for air, $n = 1.33$ for water, $n = 1.36$ for ethanol and $n = 1.496$ for toluene.

References

(1) Loudon, R. *The Quantum Theory of Light*; Oxford University Press, 2000.

(2) Shankar, R. *Principles of quantum mechanics*; Springer: New York, NY, 2012.

(3) McHale, J. L. *Molecular spectroscopy, second edition*; CRC Press, 2017.

(4) Novotny, L.; Hecht, B. *Principles of nano-optics*, 2nd ed.; Cambridge University Press: Cambridge, England, 2012.

(5) Stranius, K.; Hertzog, M.; Börjesson, K. Selective manipulation of electronically excited states through strong light–matter interactions. *Nat. Commun.* **2018**, *9*.

(6) Girard, C.; Martin, O. J. F.; Lévèque, G.; des Francs, G. C.; Dereux, A. Generalized bloch equations for optical interactions in confined geometries. *Chem. Phys. Lett.* **2005**, *404*, 44–48.

(7) Ciracì, C.; Jurga, R.; Khalid, M.; Della Sala, F. Plasmonic quantum effects on single-emitter strong coupling. *Nanophotonics* **2019**, *8*, 1821–1833.

(8) Gonçalves, P. A. D.; Christensen, T.; Rivera, N.; Jauho, A.-P.; Mortensen, N. A.; Soljačić, M. Plasmon-emitter interactions at the nanoscale. *Nat. Commun.* **2020**, *11*, 366.

(9) Kern, A. M.; Martin, O. J. F. Pitfalls in the Determination of Optical Cross Sections From Surface Integral Equation Simulations. *IEEE Transactions on Antennas and Propagation* **2010**, *58*, 2158–2161.

(10) Johnson, P. B.; Christy, R. W. Optical Constants of the Noble Metals. *Phys. Rev. B* **1972**, *6*, 4370–4379.

(11) Dorfs, D.; Härtling, T.; Miszta, K.; Bigall, N. C.; Kim, M. R.; Genovese, A.; Falqui, A.; Povia, M.; Manna, L. Reversible tunability of the near-infrared valence band plasmon resonance in Cu(2-x)Se nanocrystals. *J. Am. Chem. Soc.* **2011**, *133*, 11175–11180.

(12) West, P. R.; Ishii, S.; Naik, G. V.; Emani, N. K.; Shalaev, V. M.; Boltasseva, A. Searching for better plasmonic materials. *Laser Photon. Rev.* **2010**, *4*, 795–808.

(13) Naik, G. V.; Kim, J.; Boltasseva, A. Oxides and nitrides as alternative plasmonic materials in the optical range [Invited]. *Opt. Mater. Express* **2011**, *1*, 1090.

(14) Manthiram, K.; Alivisatos, A. P. Tunable localized surface plasmon resonances in tungsten oxide nanocrystals. *J. Am. Chem. Soc.* **2012**, *134*, 3995–3998.

(15) Gordon, T. R.; Paik, T.; Klein, D. R.; Naik, G. V.; Caglayan, H.; Boltasseva, A.; Murray, C. B. Shape-dependent plasmonic response and directed self-assembly in a new semiconductor building block, indium-doped cadmium oxide (ICO). *Nano Lett.* **2013**, *13*, 2857–2863.

(16) Ye, X.; Fei, J.; Diroll, B. T.; Paik, T.; Murray, C. B. Expanding the spectral tunability of plasmonic resonances in doped metal-oxide nanocrystals through cooperative cation-anion codoping. *J. Am. Chem. Soc.* **2014**, *136*, 11680–11686.

(17) Naik, G. V.; Shalaev, V. M.; Boltasseva, A. Alternative plasmonic materials: beyond gold and silver. *Adv. Mater.* **2013**, *25*, 3264–3294.
